# Factors related to increased alcohol misuse by students compared to non-students during the first Covid-19 lockdown in France: the Confins study

**DOI:** 10.1186/s12889-024-18182-w

**Published:** 2024-02-29

**Authors:** Shérazade Kinouani, Mélissa Macalli, Julie Arsandaux, Ilaria Montagni, Nathalie Texier, Stéphane Schück, Christophe Tzourio

**Affiliations:** 1 Univ. Bordeaux, Inserm, Bordeaux Population Health Research Center, Team HEALTHY, UMR1219, Bordeaux, 33000 France; 2 Department of General Practice, University of Bordeaux, 146 Rue Léo Saignat, Bordeaux, 33000 France; 3 Nantes Université, Univ Angers, Laboratoire de Psychologie Des Pays de La Loire, LPPL, UR 4638, Nantes, F-44000 France; 4 Kappa Santé, 4 Rue de Cléry, Paris, 75002 France; 5 Kap Code, 28 Rue d’Enghien, Paris, 75010 France

**Keywords:** Alcohol-related disorders, Binge-drinking, Covid-19, Lockdown, Students

## Abstract

**Background:**

The closure of bars and lockdowns related to the Covid-19 pandemic changed alcohol use levels in France during the spring of 2020. We wondered whether this sudden cessation of social interactions impacted students more than non-students and what factors specific to students would explain the increase in alcohol misuse. The aims of this study were to compare self-reported changes in alcohol misuse (alcohol intake and binge-drinking frequency) during the first Covid-19 lockdown from March 17 to May 10, 2020, between French students and non-students and describe factors associated with this alcohol misuse in each subgroup.

**Methods:**

Data collected in the Confins study from April 8 to May 10, 2020, were used in cross-sectional analyses stratified by student status. Multiple logistic regression was performed to estimate the association between self-reported increase in alcohol intake or binge-drinking frequency (at least six drinks of alcohol on one occasion) and demographic, socioeconomic, and clinical factors, as well as conditions associated with the Covid-19 pandemic. The population-attributable fraction was then used to estimate the contribution of identified risk factors to increased alcohol misuse in students and non-students.

**Results:**

Among both students and non-students, a self-reported decrease or no change in alcohol intake or binge-drinking was more common than an increase. However, the risk factors explaining an increase in alcohol intake differed among students (≥ 25 years old, not working or studying in the health field, and having suicidal ideation during the last 7 days) and non-students (having a medical diagnosis of mental disorders). The risk factors explaining an increase in binge-drinking frequency were similar in the two subgroups (being a tobacco smoker before lockdown and not practicing any physical activity during the last 7 days), except suicidal thoughts, which was a risk factor for alcohol misuse specific to students.

**Conclusions:**

These results highlight the vulnerability of certain French students to alcohol misuse and the necessity of combining both mental health and substance use-related screening in the student population.

## Background

The SARS-CoV-2 virus rapidly spread around the world from late 2019, leading the World Health Organization (WHO) to announce pandemic status for Covid-19 on March 11, 2020 [1]. In Europe, different measures varying by country were quickly put in place to control virus transmission after this announcement [2]. These measures often included lockdown, physical distancing, and reduction of social activities. Rather than increasing, Europeans seemed to have reduced their alcohol use in both quantity and frequency in the first months of 2020 [3–7]. This was explained by the sudden and prolonged cessation of alcohol sales in bars and restaurants, which was not compensated by an increase in alcohol sales online or by retail stores [3]. A study carried out within an online panel representative of the French adult population reported that 64.8% of this population had not changed their alcohol intake, whereas 24.4% had reduced their alcohol intake and 10.7% had increased their intake during the first Covid-19 lockdown [8]. A study in another representative sample from France found similar results during the same period [9]. European students seemed to follow the same trend of stable or reduced alcohol misuse during the spring of 2020 [10–13]. In a study of more than 17,000 European students, an overall reduction in their alcohol use was found during the Covid-19 pandemic (March 2020 to April 2021) compared to the pre-pandemic period [10]. Among 3,671 students surveyed at one university in northwestern France, the proportion of binge-drinking fell sharply during the first lockdown [11]. In a few studies, male gender, not living with parents, and reporting depressive symptoms were associated with an increased frequency of binge-drinking among the student population during the spring of 2020 [11, 13, 14]. The factors explaining an increase in alcohol misuse among students during this health crisis are beginning to be identified, but it is not clear whether they are similar to those explaining an increase among non-students.

In 2016, France was among the countries that consumed the most alcohol in the world, ranking sixth among the 34 countries of the Organization for Economic Co-operation and Development (OECD) based on the total per capita alcohol consumption [15]. The first Covid-19 lockdown in France took place from March 17 to May 10, 2020. Restaurants, bars, schools, universities, and all administrative services stopped during this period [16]. Cultural, professional, and sports gatherings were prohibited. It was possible to leave home (to go shopping, go to the doctor, or recreation) at certain times of the day provided the individual had a certificate justifying the reason for the trip. Only essential workers were able to go to their workplaces. For all others, teleworking was encouraged. If it was no longer possible to consume alcohol in bars and restaurants, its purchase was authorized in essential shops or alcohol retailers during limited time slots. Nothing forbade this consumption at home, but it was forbidden to form crowds outdoors to consume alcohol in several French cities. There have been three lockdowns related to Covid-19 in France. The lockdown from March to May 2020 was the first and strictest of the three. To the best of our knowledge, no data are available comparing alcohol use in students and non-students among French people during this first lockdown.

A high level of stress is frequently reported among college students and is related to their adaptation to a new environment, academic workload, and pressure to succeed [17, 18]. Freshmen appear particularly vulnerable [17, 19–21]. College students also have higher levels of alcohol use or binge-drinking and are more likely to misuse alcohol than adults of the same age [22–26]. This seems to be favored by meeting new drinking peers at university, reaching the age of majority, the distance from the parental home, lack of family obligations, and a greater general tolerance toward student alcohol consumption [27, 28]. We wondered if students were more greatly impacted by an increase in alcohol misuse during the beginning of Covid-19 crisis and what factors specific to students would explain this. We hypothesized that the sustainable cessation of social activities would have reduced the consumption by students more than that of non-students. Nevertheless, those who increased their alcohol use during this period probably had risk factors that deserve to be identified. We analyzed two different outcomes in relation to alcohol misuse: 1) a classical quantity of alcohol per week in order to quantify the overall amount of alcohol consumed; and 2) binge drinking as it is a particular mode of consumption very prevalent among young adults and because its determinants and consequences may not overlap with the sheer quantity of alcohol consumed regularly. This appeared to be an interesting mode of use to study because it involves consumption in large quantities over a single episode. This pattern is more common than daily alcohol consumption among French young adults [29]. Therefore, the main objective of this study was to compare the self-reported changes in alcohol intake and binge-drinking frequency in French students and non-students during the first Covid-19 lockdown. The secondary objective was to describe factors associated with an increase in alcohol intake and binge-drinking frequency during the same period in each subgroup.

## Methods

### Data source, study design, and sample

Data came from an online observational study: the Confins cohort [30]. This prospective population-based study addressed the emotional, psychological, and behavioral impact of the Covid-19 outbreak among adults living in France. Participants were recruited by advertisements on social networks (professional or academic) and press communications. To be included, subjects had to be aged ≥ 18 years, understand French, and be confined in France (i.e., living in France and having one's movements restricted by government measures linked to management of the Covid-19 outbreak). Data were collected in the Confins cohort from April 2020 until January 2022, but only the data collected at baseline during the first lockdown were used for these cross-sectional analyses (from April 8 to May 10, 2020). Subjects for whom information on gender, age, and student status was missing were excluded (*n* = 49). For changes in alcohol intake during the 7 days preceding inclusion, we excluded those with missing data (*n* = 341) or who declared no habitual use of alcohol during lockdown (*n* = 564). For binge-drinking frequency during the last 7 days, we also excluded those with missing data (*n* = 1244) or who declared no habitual practice of binge-drinking (*n* = 116).

### Outcomes of alcohol misuse during lockdown

Two self-reported outcomes were analyzed separately: alcohol intake and binge-drinking frequency. First, the change in alcohol intake was explored by asking, "During the last seven days, how has your alcohol intake changed?" Second, the change in binge-drinking frequency was explored with the question, "If you drink six drinks of alcohol on one occasion and in a short time, has it happened more frequently since lockdown?" This last question was only asked of those declaring they consumed alcohol at least once a month during the past year and who also experimented with binge-drinking during that time.

For each question, participants had four response options: no usual use, no change, decreased use, or increased use. The participants declaring "no usual use" during the last 7 days were excluded from all analyses, and each outcome was built and studied as two binary variables in two sets: increased use *versus* no change, and then increased use *versus* favorable use (defined as decreased use or no change).

### Covariates: demographic, socioeconomic, and clinical characteristics

Several characteristics identified as potential risk or confounding factors in relation to alcohol consumption were studied: age in categories (18—24 years or ≥ 25 years); gender; living with a partner (living as a couple, married or not); student status (being enrolled in an institution of higher education in France); working or studying in the health field; self-rated economic situation during childhood or adolescence and current main income source (collected only among students); medical diagnosis of mental disorders; smoking status in the past year; alcohol use disorder (AUD) in the past year. AUD in the past year was only collected in those who declared having drunk alcohol at least once a month in the past year and was defined according to the Alcohol Use Disorders Identification Test-Consumption (AUDIT-C) [31, 32]. AUD corresponded to an AUDIT-C score ≥ 3 in women or ≥ 4 in men. Regarding mental health, we explored physical activity and suicidal thoughts during the last 7 days. The presence of anxiety symptoms was also assessed with the Generalized Anxiety Disorder 7 items (GAD-7) scored as 0–4 (none), 5–9 (mild), 10–14 (moderate), and 15–21 (severe) [33]. Depressive symptoms were assessed with the Patient Health Questionnaire 9 items (PHQ-9) scored as 0–4 (none), 5–9 (mild), 10–14 (moderate), 15–19 (moderately severe), and 20–27 (severe) [34]. A high level of anxiety was defined as a GAD-7 score ≥ 10 and a high level of depressive symptoms as a PHQ-9 score ≥ 10. Finally, conditions associated with the Covid-19 pandemic were also explored in relation to alcohol misuse: being confined alone and being confined in a geographic zone with an excess of deaths between March 30 and April 5. The excess deaths were determined by the French public health agency *Santé Publique France* by a standardized indicator comparing the weekly observed number of deaths to the expected number estimated from a statistical model used by 24 European countries in the EuroMOMO (European monitoring of excess mortality for public health action) consortium [35]. We also studied whether presenting with symptoms suggestive of Covid-19 or having a Covid-19 diagnosis by a physician, in the presence or absence of an antigen test, affected alcohol misuse.

### Statistical analyses

We described the total sample and both the student and non-student subgroups. Categorical variables were described as frequencies and percentages and continuous variables as medians and interquartile ranges (IQRs). We compared the change in alcohol intake and binge-drinking frequency in the last 7 days (increased alcohol use *versus* no change and then *versus* favorable use) in students and non-students using chi-square test. Next, we focused on factors associated with an increase in alcohol intake or binge-drinking frequency using multivariate logistic regression models. Each logistic regression analysis compared increased use to favorable use. For each model, odds ratios (ORs) were provided with their 95% confidence intervals (CIs). All identified covariates were tested as potential confounding factors. The covariate identification consisted of retaining from the data available in the Confins cohort those which appeared relevant according to the existing literature. These identified covariates were then statistically selected for inclusion in models. For this, we performed bivariate analyses and tested interactions between factors. Covariates and interactions were retained in the initial multivariate model when they were associated with the outcome with significance at a *P* value of 0.25 or below. For an interaction, if *p* < 0.25, variables and interaction terms were also entered into the initial multivariate model. PHQ-9 ≥ 10, GAD-7 ≥ 10, and suicidal thoughts were collinear. Even when several of these collinear variables were associated with outcome in the bivariate analysis at the *p* < 0.25 threshold, only one of them was introduced into the initial multivariate logistic regression model. We then performed backward stepwise selection to obtain a final multivariate model (model 1), with a significant *p* < 0.05 for each model. We forced age in classes and gender in model 1 with the exception of those explaining the association between the increase in binge-drinking frequency among non-students. Age in classes was not forced in this last model; it did not converge in the bivariate analysis due to the small sample size. All multivariate logistic regression analyses were stratified by student status.

We finally calculated the rise in the population-attributable fraction (PAF) for each identified risk factor for an increase in alcohol intake or binge-drinking frequency using the following equation:

$$\mathrm{PAF}=\lbrack\mathrm p^\ast(\text{AOR}-1)\rbrack/\lbrack1+(\mathrm p^\ast(\text{AOR}-1)\rbrack$$

where p was the prevalence of the risk factor in our sample and AOR was the adjusted odds ratio related to the association between the risk factor and the outcome [36]. We used the AORs of model 1 to determine the PAF in students and non-students.

All analyses were carried out in SAS version 9.4 (SAS Institute Inc.), and all tests were two-tailed.

## Results

### Change in alcohol intake during the last seven days

**Fig. 1:**
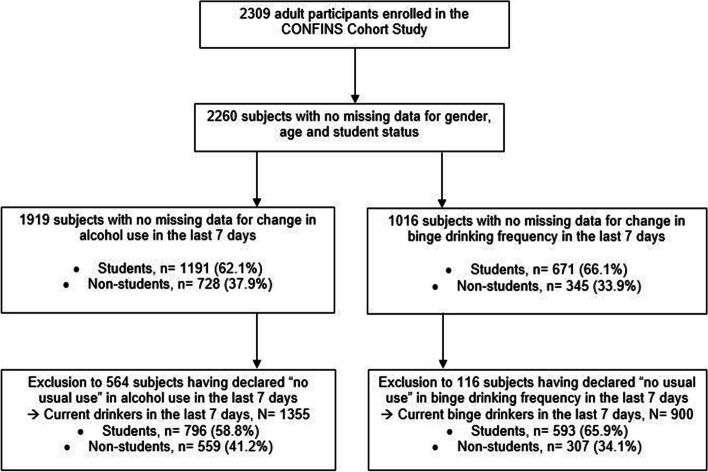
Flow chart of participants in the Confins cohort, France

Among the 2309 persons eligible for the study, 1919 answered the question about changes in their alcohol intake during the last 7 days of lockdown, but 1355 of them were retained for analysis (Fig. 1): 58.8% were students and 41.2% non-students. In this sample, almost four in five participants were women and 40.0% were health students or health workers (Table 1).

**Table 1:** Descriptive characteristics of current drinkers^a^

Characteristics	Total sample *N* = 1355		Students *N* = 796		Non-students *N* = 559		*p*-value
	**n**	**%**	**n**	**%**	**n**	**%**	
Median age (IQR)	25.1 (22.5–31.9)		23.0 (21.4–24.9)		34.6 (27.2–52.1)		< 0.0001
≥ 25 years (ref. 18–24 years)	691	51.0	196	24.6	495	88.5	< 0.0001
Men	316	23.3	160	20.1	156	27.9	0.0008
Living with a partner (ref. single or other)	793	58.5	400	50.2	393	70.3	< 0.0001
Working (or studying) in the health field (ref. no working or studying in the health field)	542	40.0	318	39.9	224	40.1	0.9641
Self-rated economic situation during childhood or adolescence among students. MD = 57							/
• Very comfortable to comfortable	/	/	489	66.2	/	/	
• Suitable	/	/	195	26.4	/	/	
• Difficult to very difficult	/	/	55	7.4	/	/	
Current main source of income among students. MD = 57							/
• Family	/	/	400	54.1	/	/	
• Scholarship	/	/	132	17.9	/	/	
• Paid activities	/	/	158	21.4	/	/	
• Others (student loan, thrift, etc.)	/	/	49	6.6	/	/	
Medical diagnosis of mental disorders during lifetime (ref. no medical diagnosis of mental disorders)	295	21.8	175	22.0	120	21.5	0.8201
Tobacco smoking in the past 12 months (ref. former smoking or no smoking)	365	26.9	236	29.6	129	23.1	0.0073
Having drinking alcohol at least once a month in the past 12 months (ref. never or less than once a month)	1266	93.4	733	92.1	533	95.3	0.0170
Alcohol use disorder^b^ in the past 12 months (ref. no alcohol use disorder). MD = 2	943	74.6	577	78.8	366	68.8	< 0.0001
Having been affected (formerly or currently) by Covid-19, with or without diagnostic test (ref. no having affected by Covid-19)	119	8.8	74	9.3	45	8.0	0.4249
Lockdown alone (ref. no being alone during lockdown)	212	15.6	127	15.9	85	15.2	0.7087
Being confined in a French zone with an excess of deaths (ref. no confined in risk zone). MD = 14	519	38.7	227	28.8	292	52.7	< 0.0001
Physical activity in the last seven days (ref. no physical activity)	1151	84.9	664	83.4	487	87.1	0.0606
Suicidal thoughts in the last seven days (ref. no suicidal thoughts)	131	9.7	93	11.7	38	6.8	0.0027
PHQ-9 ≥ 10^c^	366	27.0	270	33.9	96	17.2	< 0.0001
GAD-7 ≥ 10^d^	294	21.7	212	26.6	82	14.7	< 0.0001
Self-reported changes in alcohol intake during the last seven days							< 0.0001
• No change	514	37.9	252	31.7	262	46.9	
• Decreased use	537	39.6	390	49.0	147	26.3	
• Increased use	304	22.5	154	19.3	150	26.8	

*IQR* Interquartile range, *MD* Missing data

^a^Analyses excluded 564 participants declaring "no usual use" in response to the question, "During the last seven days, how has your alcohol intake changed?"

^b^Alcohol use disorder was only explored in those who declared having drank alcohol at least once a month in the past 12 months. Alcohol use disorder was defined according to AUDIT-C score ≥ 3 in women and ≥ 4 in men

^c^PHQ-9: Patient Health Questionnaire 9 items scored as 0–4 (none), 5–9 (mild), 10–14 (moderate), 15–19 (moderately severe), 20–27 (severe)

^d^GAD-7: Generalized Anxiety Disorder 7 items scored as 0–4 (none), 5–9 (mild), 10–14 (moderate), 15–21 (severe)

AUD or tobacco smoking in the past year were more common among students than non-students. High levels of anxiety or depression and suicidal thoughts during the last 7 days were also more frequently reported by students (Table 1).

Compared to favorable use, increased alcohol intake was less common among students than non-students (Table 2).

**Table 2:** Comparison of changes in alcohol intake according to student status among current drinkers^a^

	**Students**		**Non-students**		***P* value**
	**n**	**%**	**n**	**%**	
Increased use *versus* no change, *N* = 818					0.6522
• Increased use	154	37.9	150	36.4	
• No change	252	62.1	262	63.6	
Increased use *versus* “no change or decreased use”, *N* = 1355					0.0011
• Increased use	154	19.4	150	26.8	
• No change or decreased use	642	80.6	409	73.2	

^a^Analyses excluded 564 participants declaring "no usual use" in response to the question: "During the last seven days, how has your alcohol intake changed?"

Table 3 summarizes factors associated with an increase in alcohol intake during the last 7 days. These factors differed according to student status.

**Table 3:** Factors associated with an increase in alcohol intake according to student status

	**Students**		**Non-students**		
	**Bivariate analysis**^a^	**Model 1**^b^	**Bivariate analysis**^a^		**Model 1**^c^
**Characteristics**	**OR (95% CI)**	**AOR (95% CI)**	**OR (95% CI)**		**AOR (95% CI)**
≥ 25 years (ref. 18–24 years)	1.63 (1.11–2.34)*	1.87 (1.25–2.79)**	1.02 (0.56–1.83)		0.95 (0.52–1.73)
Men (ref. women)	1.11 (0.72–1.70)	1.13 (0.73–1.76)	1.37 (0.91–2.06)		1.43 (0.95–2.16)
Living with a partner (ref. single or other)	1.28 (0.90–1.82)	/	1.28 (0.84–1.95)		/
Studying in the health field (ref. no studying in the health field)	0.69 (0.48–1.01)	0.63 (0.43–0.93)*	/		/
Self-rated economic situation during childhood or adolescence among students					
• Very comfortable to comfortable	Ref	/	/		/
• Suitable	0.94 (0.61–1.45)	/	/		/
• Difficult to very difficult	1.93 (1.04–3.57)	/	/		/
Current main source of income among students					
• Family	Ref	/	/		/
• Scholarship	1.18 (0.71–1.97)	/	/		/
• Paid activities	1.60 (1.02–2.51)*	/	/		/
• Others (student loan, thrift, etc.)	2.23 (1.15–4.33)*	/	/		/
Medical diagnosis of mental disorders during lifetime (ref. no medical diagnosis of mental disorders)	/	/	1.65 (1.07–2.55)*		1.70 (1.10–2.64)*
Tobacco smoking in the past 12 months (ref. former smoking or no smoking)	1.47 (1.01–2.12)*	/	/		/
Alcohol use disorder^d^ in the past 12 months (ref. no alcohol use disorder)	/	/	1.37 (0.90–2.10)		/
Having been affected (formerly or currently) by Covid-19, with or without diagnostic test (ref. no having affected by Covid-19)	/	/	1.56 (0.82–2.97)		/
Lockdown alone (ref. no being alone during lockdown)	/	/	/		/
Being confined in a French zone with an excess of deaths (ref. no confined in risk zone)	/	/	/		/
Physical activity in the last seven days (ref. no physical activity)	0.70 (0.45–1.10)	/	/		/
Suicidal thoughts in the last seven days (ref. no suicidal thoughts)	1.74 (1.07–2.85)*	1.74 (1.06–2.88)*	2.10 (1.07–4.12)*		/

*AOR* Adjusted odds ratio, *OR* Odds ratio, *Ref* Reference, *CI* Confidence interval

^*^p < 0.05, **p ≤ 0.01, ***p ≤ 0.001

^a^Bivariate analysis: tested potential factors were categorized age; gender; living with partner; working (or studying) in the health field; medical diagnosis of mental disorders during lifetime; smoking status in the past year; alcohol use disorder in the past year; having been affected (formerly or currently) by Covid-19, with or without a diagnostic test; lockdown alone; being confined in a French zone with an excess of deaths; physical activity in the last 7 days; suicidal thoughts in the last 7 days; PHQ-9 ≥ 10; GAD-7 ≥ 10; self-rated economic situation during childhood or adolescence; and current main income source (collected only among students). Only those associated with the outcome with significance at 0.25 were included in the initial multivariate logistic regression model and are presented in Table 3. PHQ-9 ≥ 10, GAD-7 ≥ 10, and suicidal thoughts were collinear. Even when these variables were associated with outcome in the bivariate analysis at the *p* < 0.25 threshold, none of the three were introduced into the initial multivariate logistic regression models. Only the variable “suicidal thoughts” was then kept

^b^Model 1 (*n* = 796): multivariate logistic regression model with backward stepwise selection by forcing age in classes and gender in the models if necessary on complete data (no missing data)

^c^Model 1 (*n* = 559): multivariate logistic regression model with backward stepwise selection by forcing age in classes and gender into the models if necessary on complete data (no missing data)

^d^Alcohol use disorder was only explored in those who declared having drank alcohol at least once a month in the past 12 months. It was defined according to AUDIT-C score ≥ 3 in women and ≥ 4 in men

Being ≥ 25 years old (PAF = 17.6%), not working or studying in the health field (PAF = 25.5%), and having suicidal thoughts during the last 7 days (PAF = 8.0%) were positively associated with increased alcohol intake in the student subgroup. Health students were less likely to report increased alcohol intake than students in other fields. In the non-student population, declaring a medical diagnosis of mental disorders (PAF = 13.1%) was positively associated with increased alcohol intake.

### Change in binge-drinking frequency during the last seven days

Among the 1016 participants with no missing data regarding binge-drinking frequency during the last 7 days of lockdown, 900 were retained for analysis (Fig. 1). In this subgroup, 74.3% were women. The student subgroup also had more subjects aged 18–24 years, reported more suicidal thoughts, and more commonly had high PHQ-9 or GAD-7 scores than non-students (Table 4).

**Table 4:** Descriptive characteristics of current binge drinkers^a^

Characteristics	Total sample *N* = 900		Students *N* = 593		Non-students *N* = 307		*p*-value
	**n**	**%**	**n**	**%**	**n**	**%**	
Median age (IQR)	24.3 (22.2–27.9)		23.0 (21.3–24.6)		30.4 (26.3–43.4)		< 0.0001
≥ 25 years (ref. 18–24 years)	393	43.7	131	22.1	262	85.3	< 0.0001
Men	231	25.7	127	21.4	104	33.9	< 0.0001
Living with a partner (ref. single or other)	493	54.8	294	49.6	199	64.8	< 0.0001
Working (or studying) in the health field (ref. no working or studying in the health field)	388	43.1	266	44.9	122	39.7	0.1417
Self-rated economic situation during childhood or adolescence among students. MD = 44							/
• Very comfortable to comfortable	/	/	369	67.2	/	/	
• Suitable	/	/	146	26.6	/	/	
• Difficult to very difficult	/	/	34	6.2	/	/	
Current main source of income among students. MD = 44							/
• Family	/	/	308	56.1	/	/	
• Scholarship	/	/	87	15.8	/	/	
• Paid activities	/	/	120	21.9	/	/	
• Others (student loan, thrift, etc.)	/	/	34	6.2	/	/	
Medical diagnosis of mental disorders during lifetime (ref. no medical diagnosis of mental disorders)	196	21.8	135	22.8	61	19.9	0.3183
Tobacco smoking in the past 12 months (ref. former smoking or no smoking)	304	33.8	207	34.9	97	31.6	0.3194
Alcohol use disorder^b^ in the past 12 months (ref. no alcohol use disorder)	820	91.1	541	91.2	279	90.9	0.8605
Having been affected (formerly or currently) by Covid-19, with or without diagnostic test (ref. no having affected by Covid-19)	90	10.0	58	9.8	32	10.4	0.7606
Lockdown alone (ref. no being alone during lockdown)	146	16.2	95	16.0	51	16.6	0.8193
Being confined in a French zone with an excess of deaths (ref. no confined in risk zone). MD = 6	330	36.9	179	30.2	151	50.0	< 0.0001
Physical activity in the last seven days (ref. no physical activity)	763	84.8	498	84.0	265	86.3	0.3543
Suicidal thoughts in the last seven days (ref. no suicidal thoughts)	98	10.9	76	12.8	22	7.2	0.0099
PHQ-9 ≥ 10^c^	265	29.4	207	34.9	58	18.9	< 0.0001
GAD-7 ≥ 10^d^	199	22.1	150	25.3	49	16.0	0.0014
Self-reported changes in binge-drinking frequency during the last seven days							< 0.0001
• No change	381	42.3	226	38.1	155	50.5	
• Decreased use	470	52.2	343	57.8	127	31.4	
• Increased use	49	5.5	24	4.1	25	8.1	

*IQR* Interquartile range, *MD* Missing data

^a^Analyses excluded 116 participants declaring “no usual use” in response to the question, "If you drink six drinks of alcohol on one occasion and in a short time, has it happened more frequently since lockdown?"

^b^Alcohol use disorder was only explored in those who declared having drank alcohol at least once a month in the past 12 months. It was defined according to AUDIT-C score ≥ 3 in women and ≥ 4 in men

^c^PHQ-9: Patient Health Questionnaire 9 items scored as 0–4 (none), 5–9 (mild), 10–14 (moderate), 15–19 (moderately severe), 20–27 (severe)

^d^GAD-7: Generalized Anxiety Disorder 7 items scored as 0–4 (none), 5–9 (mild), 10–14 (moderate), 15–21 (severe)

Compared to favorable use, students less often reported having increased their frequency of binge-drinking than non-students (Table 5).

**Table 5:** Comparison of changes in binge-drinking frequency^a^ according to student status among current binge drinkers^b^

	**Students**		**Non-students**		***P* value**
	**n**	**%**	**n**	**%**	
Increased use *versus* no change, *N* = 430					0.1673
• Increased use	24	9.6	25	13.9	
• No change	226	90.4	155	86.1	
Increased use *versus* “no change or decreased use”, *N* = 900					0.0102
• Increased use	24	4.1	25	8.1	
• No change or decreased use	569	95.9	282	91.9	

^a^The change in binge-drinking frequency during lockdown was explored in those who drank alcohol at least once a month and had previously experienced binge-drinking during the past 12 months

^b^Analyses excluded 116 participants declaring “no usual use” in response to the question, "If you drink six drinks of alcohol on one occasion and in a short time, has it happened more frequently since lockdown?"

Among students, an increase in the frequency of binge-drinking was positively linked to being a smoker in the past year (PAF = 41.0%) and declaring suicidal thoughts (PAF = 29.3%; Table 6). Among non-students, an increase in binge-drinking frequency was associated with being a smoker during the past year only among men (PAF = 76.8%). Not practicing any physical activity in the past 7 days increased the likelihood of reporting an increase in binge-drinking frequency among both students (PAF = 21.8%) and non-students (PAF = 28.4%).

**Table 6:** Factors associated with an increase in binge-drinking frequency^a^ according to student status

	**Students**		**Non-students**	
	**Bivariate analysis**^b^	**Model 1**^c^	**Bivariate analysis**^b^	**Model 1**^d^
**Characteristics**	**OR** **(95% CI)**	**AOR** **(95% CI)**	**OR** **(95% CI)**	**AOR** **(95% CI)**
≥ 25 years (ref. 18–24 years)	1.81 (0.76–4.34)	1.80 (0.71–4.54)	/	/
Men (ref. women)	1.23 (0.48–3.18)	1.31 (0.48–3.55)	2.27 [0.99–5.80]	/
Living with a partner (ref. single or other)	/	/	/	/
Studying in the health field (ref. no studying in the health field)	0.49 (0.20–1.21)	/	/	/
Self-rated economic situation during childhood or adolescence among students				
• Very comfortable to comfortable	Ref	/	/	/
• Suitable	0.66 (0.22–2.04)	/	/	/
• Difficult to very difficult	3.15 (0.98–10.08)	/	/	/
Current main source of income among students				
• Family	Ref	/	/	/
• Scholarship	0.81 (0.23–2.91)	/	/	/
• Paid activities	0.58 (0.16–2.08)	/	/	/
• Others (student loan, thrift, etc.)	3.03 (0.93–9.87)	/	/	/
Medical diagnosis of mental disorders during lifetime (ref. no medical diagnosis of mental disorders)	1.74 (0.73–4.16)	/	2.49 (1.04–5.94)*	/
Tobacco smoking in the past 12 months (ref. former smoking or no smoking)	3.27 (1.41–7.61)**	2.99 (1.25–7.13)*	3.05 (1.33–7.00)**	
• Men			/	13.30 (3.21–55.10) ***
• Women			/	1.58 (0.33–4.08)
Alcohol use disorder^e^ in the past 12 months (ref. no alcohol use disorder)	/	/	/	/
Having been affected (formerly or currently) by Covid-19, with or without diagnostic test (ref. no having affected by Covid-19)	/	/	3.11 (1.14–8.47)*	/
Lockdown alone (ref. no being alone during lockdown)	2.25 (0.91–5.59)	/	/	/
Being confined in a French zone with an excess of deaths (ref. no confined in risk zone)	/	/	/	/
Physical activity in the last seven days (ref. no physical activity)	0.30 (0.13–0.70)**	0.36 (0.15–0.89)*	0.24 (0.10–0.58)**	0.26 (0.10–0.68)**
Suicidal thoughts in the last seven days (ref. no suicidal thoughts)	4.50 (1.89–10.67)***	4.24 (1.67–10.64)**	/	/
PHQ-9 ≥ 10^f^			2.67 (1.12–6.40)*	/

*AOR* Adjusted odds ratio, *OR* Odds ratio, *Ref* Reference, *CI* Confidence interval

^*^p < 0.05, **p ≤ 0.01, ***p ≤ 0.001

^a^The change in binge-drinking during lockdown was explored in those who drank alcohol at least once a month and had previously experienced binge-drinking during the past 12 months

^b^Bivariate analysis: tested potential factors were categorized age; gender; living with partner; working (or studying) in the health field; medical diagnosis of mental disorders during lifetime; smoking status in the past year; alcohol use disorder in the past year; having been affected (formerly or currently) by Covid-19, with or without a diagnostic test; lockdown alone; being confined in a French zone with an excess of deaths; physical activity in the last 7 days; suicidal thoughts in the last 7 days; PHQ-9 ≥ 10; GAD-7 ≥ 10; self-rated economic situation during childhood or adolescence; and current main income source (collected only among students). Only those associated with the outcome with significance at 0.25 were included into the initial multivariate logistic regression model and are presented in Table 6. PHQ-9 ≥ 10, GAD-7 ≥ 10, and suicidal thoughts were collinear. Even when these variables were associated with outcome in the bivariate analysis at the *p* < 0.25 threshold, none of the three were introduced into the initial multivariate logistic regression models. The variable “suicidal thoughts” was kept in the initial logistic regression model among students, PHQ-9 ≥ 10 was kept in among non-students

^c^Model 1 (*n* = 593): multivariate logistic regression model with backward stepwise selection by forcing age in classes and gender in the models on complete data (no missing data)

^d^Model 1 (*n* = 307): multivariate logistic regression model with backward stepwise selection by forcing gender into the models on complete data (no missing data). The variable age in classes was not forced in this model; it did not converge in the bivariate analysis due to the small sample size. An interaction between gender and smoking status was significant at the *p* < 0.25 threshold and introduced into the multivariate logistic regression model

^e^Alcohol use disorder was only explored in those who declared having drank alcohol at least once a month in the past 12 months. It was defined according to AUDIT-C score ≥ 3 in women and ≥ 4 in men

^f^PHQ-9: Patient Health Questionnaire 9 items scored as 0–4 (none), 5–9 (mild), 10–14 (moderate), 15–19 (moderately severe), 20–27 (severe)

## Discussion

During the first Covid-19 lockdown in France, a self-reported reduction or no change in alcohol intake or binge-drinking frequency was more common than an increase. The risk factors explaining an increase in alcohol intake differed among students (≥ 25 years of age, not working or studying in the health field, and having suicidal ideation during the last 7 days) and non-students (having a medical diagnosis of mental disorders). Being a tobacco smoker before lockdown and not practicing any physical activity during the last 7 days were common risk factors for an increased frequency of binge-drinking among both students and non-students. Nevertheless, suicidal thoughts were a specific risk factor for an increased frequency of binge-drinking among students.

Our results regarding the change in alcohol misuse during the spring of 2020 are consistent with other reports in the French population [8, 9, 37], although the information on the French student population is very limited [11, 38, 39]. Alcohol misuse was less frequent among students than non-students during the spring of 2020. This corroborated our hypothesis of a decrease in alcohol use among students in connection with the drastic reduction in social activities during the first Covid-19 lockdown. Indeed, alcohol use by young adults occurs mainly during social events [40, 41]. However, this remains a hypothesis as we did not collect data on changes in social activities in our study.

Based on the PAF, age ≥ 25 years is a risk factor for increased alcohol intake among students during this period. This pattern could have several explanations. The closure of bars and nightclubs, the ban on gatherings, or a return to the parental home were all potential reasons why students normally reduced their consumption during the first lockdown [42–44]. However, new drinking opportunities in private settings appeared: virtual happy hours, illegal parties, or alcohol delivery at home [45–48]. Some studies have also reported an increase in solitary alcohol use among college students during the Covid-19 pandemic [44, 48]. Therefore, we hypothesized that the alcohol use by older students would be less dependent on the closure of usual places of socialization outside the home during this period. They probably practiced more solitary use or had more means to organize virtual or physical private events where it was possible to consume alcohol because of, for example, lack of parental control, more financial resources, and living independently.

Based on the PAF, tobacco use in the past year was a major risk factor for an increase in binge-drinking frequency among students and male non-students. These results were consistent with other studies conducted during the Covid-19 pandemic [13, 49]. The association between tobacco use and binge-drinking had already been described before the Covid-19 crisis [50–54]. Moreover, preclinical studies have shown that nicotine administration increases alcohol consumption and alcohol self-administration behavior or restores alcohol-seeking in rats [55–58]. Thus, it was unsurprising to see that this relationship persisted during the Covid-19 pandemic in the event of an increase in binge-drinking frequency. We observed a gender difference in tobacco use among non-students. There was an increase in binge drinking among male smokers, but not among non-smokers. We don't have a straightforward explanation for this finding, which needs to be confirmed in other studies.

We also found a relationship between suicidal thoughts and alcohol misuse (both alcohol intake and binge-drinking frequency) in students. This association has already been described outside the context of the Covid-19 pandemic [59, 60]. Suicidal ideation is common among students [61]. Its association with alcohol misuse is probably partly mediated by mental health factors [60, 62–65]. This would imply that students could have increased alcohol use to cope with stress or manage posttraumatic stress, anxiety, or mood disorders during the Covid-19 pandemic [66–68]. Mental health symptoms may have been exacerbated among the students who were most uncertain regarding their careers following the cessation of university courses and internships [69, 70], facilitating the onset of suicidal thoughts. Finally, the feeling of loneliness induced by the sustainable cessation of social activities and lockdown conditions has also been described as a factor underlying suicidal thoughts among students, regardless of mental health disorders [71–73].

### Strengths & limitations

This study took place during the first French lockdown of the Covid-19 pandemic. During this complex period, we were able to rapidly set up a population-based study and gather a relatively large sample of individuals. Many variables were collected, especially regarding mental health and the Covid-19 outbreak. This made it possible to test the associations between our two main outcomes and several potential risk factors. Another strength was comparing the alcohol misuse of students to that of non-students.

However, this work had some limitations. Our sample is a convenience sample and, therefore, not representative of the French population at large. This explains the high proportion of women or young adults who participated in the study. Changes in alcohol intake and binge-drinking were self-reported, which is usual in this type of study, but social desirability and memorization bias may underestimate the true frequencies. Socioeconomic characteristics were only collected for the student subgroup, not allowing us to also study these factors among non-students. In addition, the AUDIT-C score was calculated only in those who drank alcohol at least once a month before the lockdown. This excluded a few subjects with AUD who consumed alcohol less than once per month before lockdown and those who initiated alcohol use during the Covid-19 crisis. We think that such individuals are infrequent and that their absence did not influence the main results. Similarly, increased binge-drinking was explored only among those who drank alcohol at least once a month and reported ever binge-drinking in the past 12 months. This led to a significant amount of missing data. Finally, the number of subjects reporting increased alcohol intake or binge-drinking frequency was relatively low. This may have generated a lack of power in the study of the factors associated with this change in alcohol misuse.

Nevertheless, the results highlight the vulnerability of French students to alcohol misuse in times of health crisis compared to non-students, particularly for those who were older, tobacco smoker, not practicing physical activity, and having suicidal ideation. These results could help to devise a better combination of mental health and substance use-related health prevention and care services in French students’ environments.

## Conclusions

Increase in alcohol misuse was both less common among students than non-students and less common than a decrease or no change in alcohol use during the first French lockdown. Factors associated with increased alcohol intake differed between students and non-students, but factors explaining an increase in binge-drinking frequency were similar in the two subgroups. However, suicidal thoughts was a risk factor for alcohol misuse specific to the student population.

## Data Availability

The datasets used and analyzed during the current study are not available from the corresponding author but can be available upon reasonable request from the data owner – Kap Code team – using the online form: https://www.confins.org/contact/.

## Abbreviations

AUD: Alcohol use disorder
AUDIT-C: Alcohol Use Disorders Identification Test-Consumption
AOR: Adjusted odds ratio
CI: Confidence interval
EuroMOMO: European Mortality Monitoring Project
GAD-7: Generalized Anxiety Disorder 7 items
IQR: Interquartile range
OECD: Organization for Economic Co-operation and Development
OR: Odds ratio
PAF: Population attributable fraction
PHQ-9: Patient Health Questionnaire 9 items
WHO: World Health Organization
