# Correction to: Factors related to increased alcohol misuse by students compared to non-students during the first Covid-19 lockdown in France: the confins study

**DOI:** 10.1186/s12889-024-18275-6

**Published:** 2024-03-12

**Authors:** Sherazade Kinouani, Melissa Macalli, Julie Arsandaux, Ilaria Montagni, Nathalie Texier, Stephane Schuck, Christophe Tzourio

**Affiliations:** 1 University of Bordeaux, Inserm, Bordeaux Population Health Research Center, Team HEALTHY, UMR1219, 33000 Bordeaux, France; 2 Department of General Practice, University of Bordeaux, 146 Rue Leo Saignat, 33000 Bordeaux, France; 3 Nantes Universite, Univ Angers, Laboratoire de Psychologie Des Pays de La Loire, LPPL, UR 4638, F-44000 Nantes, France; 4 Kappa Sante, 4 Rue de Clery, 75002 Paris, France; 5 Kap Code, 28 Rue d’Enghien, 75010 Paris, France

**Correction to**: ***BMC Public Health*24, 646 (2024).**10.1186/s12889-024-18182-w

In the original publication of this article there was a typo in Eq. 1 which was not amended during the publication process. The incorrect and correct Eq. 1 are shown in this correction article. The original article has been updated.

**Incorrect**

$$ \text{P}\text{A}\text{F} = \left[{\text{p}}^{*}\right(\text{A}\text{O}\text{R}\hspace{0.17em}-\hspace{0.17em}1\left)\right] / [1+-({\text{p}}^{*}(\text{A}\text{O}\text{R}\hspace{0.17em}-\hspace{0.17em}1)]$$

**Correct**

$$ \text{P}\text{A}\text{F} = \left[{\text{p}}^{*}\right(\text{A}\text{O}\text{R} -\hspace{0.17em}1\left)\right] / [1+({\text{p}}^{*}(\text{A}\text{O}\text{R}-1)]$$

